# Luminal breast cancer metastases and tumor arousal from dormancy are promoted by direct actions of estradiol and progesterone on the malignant cells

**DOI:** 10.1186/s13058-014-0489-4

**Published:** 2014-12-05

**Authors:** Ndiya Ogba, Nicole G Manning, Brian S Bliesner, S Kelly Ambler, James M Haughian, Mauricio P Pinto, Paul Jedlicka, Kristiina Joensuu, Päivi Heikkilä, Kathryn B Horwitz

**Affiliations:** 1 Department of Medicine, University of Colorado Anschutz Medical Campus, 12801 E. 7th Avenue, Aurora, 80045 CO USA; 2 Department of Pathology, University of Colorado Anschutz Medical Campus, 12801 E. 7th Avenue, Aurora, 80045 CO USA; 3 Department of Pathology, University of Helsinki, 12801 E. 7th Avenue, Helsinki, 00014 Finland

## Abstract

**Introduction:**

Luminal, estrogen receptor-positive (ER^+^) breast cancers can metastasize but lie dormant for years before recurrences prove lethal. Understanding the roles of estrogen (E) or progestin (P) in development of luminal metastases or in arousal from dormancy is hindered by few preclinical models. We have developed such models.

**Methods:**

Immunocompromised, ovariectomized (ovx’d) mice were intracardiac-injected with luminal or basal human breast cancer cells. Four lines were tested: luminal ER^+^PR^+^ cytokeratin 5-negative (CK5^−^) E3 and MCF-7 cells, basal ER^−^PR^−^CK5^+^ estrogen withdrawn-line 8 (EWD8) cells, and basal ER^−^PR^−^CK5^−^ MDA-MB-231 cells. Development of micrometastases or macrometastases was quantified in ovx’d mice and in mice supplemented with E or P or both. Metastatic deposits were analyzed by immunohistochemistry for luminal, basal, and proliferation markers.

**Results:**

ER^−^PR^−^ cells generated macrometastases in multiple organs in the absence or presence of hormones. By contrast, ovx’d mice injected with ER^+^PR^+^ cells appeared to be metastases-free until they were supplemented with E or E+P. Furthermore, unlike parental ER^+^PR^+^CK5^−^ cells, luminal metastases were heterogeneous, containing a significant (6% to 30%) proportion of non-proliferative ER^−^PR^−^CK5^+^ cells that would be chemotherapy-resistant. Additionally, because these cells lack receptors, they would also be endocrine therapy-resistant. With regard to ovx’d control mice injected with ER^+^PR^+^ cells that appeared to be metastases-free, systematic pathologic analysis of organs showed that some harbor a reservoir of dormant micrometastases that are ER^+^ but PR^−^. Such cells may also be endocrine therapy- and chemotherapy-resistant. Their emergence as macrometastases can be triggered by E or E+P restoration.

**Conclusions:**

We conclude that hormones promote development of multi-organ macrometastases in luminal disease. The metastases display a disturbing heterogeneity, containing newly emergent ER^−^PR^−^ subpopulations that would be resistant to endocrine therapy and chemotherapy. Similar cells are found in luminal metastases of patients. Furthermore, lack of hormones is not protective. While no overt metastases form in ovx’d mice, luminal tumor cells can seed distant organs, where they remain dormant as micrometastases and sheltered from therapies but arousable by hormone repletion. This has implications for breast cancer survivors or women with occult disease who are prescribed hormones for contraception or replacement purposes.

**Electronic supplementary material:**

The online version of this article (doi:10.1186/s13058-014-0489-4) contains supplementary material, which is available to authorized users.

## Introduction

Luminal breast cancers represent over 70% of cases [1]. At least 1% of their cells express estrogen (ER^+^) or progesterone (PR^+^) receptors or both [2], driving estrogen (E)-dependent growth. Despite progress toward early diagnoses and advances in treatment, 20% to 30% of all patients with breast cancer and 40% to 50% of patients with luminal breast cancer experience relapses that include distant metastases [3],[4]. This tends to occur within the first 5 years for patients with basal-like ER^−^PR^−^ or HER2^+^ disease and later for patients with luminal disease [5]. In one study, median 15-year distant relapse rates were 27.8% for luminal A and 42.9% for luminal B [5]. Because molecular properties of primary tumors may be preserved in metastases [6], adjuvant endocrine therapies can improve initial survival rates even in patients with advanced luminal disease [7]. Nevertheless, the survival curve for luminal disease declines steadily after 5 years, overtaking more aggressive breast cancer subtypes after about 15 years [5],[8]. Therefore, since they represent the most common forms of the disease, luminal tumors are responsible for most breast cancer deaths. Explanations for prolonged luminal tumor dormancy and their slow but inexorable recurrence and lethality remain unclear, and roles of cellular heterogeneity and hormones in this process, if any, are poorly understood.

The Women’s Health Initiative (WHI) report on postmenopausal hormone replacement therapy (HRT) showed that the risks of combined E plus progestin (P), unlike those of physiological E alone, outweighed the benefits [9]. Widespread acceptance of the WHI data led to a general decrease in HRT use. Concurrent reductions in the incidence of invasive luminal cancers indirectly validated the WHI conclusions [10]. However, explanations for the deleterious effects on the breast of physiological E and P in combination HRT remain unclear partly because hormonal effects on carcinogenesis versus proliferation are often conflated, and the term “risk” intimates that the hormones are causative. P appears to have no effect on long-term tumor growth *in vivo* [11] but expands normal adult mammary stem cells and cancer stem cells [12]–[14]. Regarding WHI, we therefore postulated that for E+P, the P component, in a non-proliferative step, reactivates cancer stem cells in pre-existing but undiagnosed, perhaps dormant, disease [15]. That said, little is known about the roles of E and P in metastasis and recurrence from dormancy.

Clinically, the major sites of luminal metastases are bone (>49%), followed by pleura/peritoneum, liver and lung (~20%), distant nodes (~14%), and brain (6%) [5]. The ER and PR status of the primary tumor may be reflected in bone metastases [5],[16], explaining the use of endocrine therapies to treat disseminated disease that is rarely reanalyzed for biomarker expression. Few solid tumor models exist for detailed studies of luminal metastases and their hormone regulation. One interesting new model uses serially transplanted patient-derived luminal tumor xenografts, three of which demonstrate E-dependent growth and retain luminal markers and gene expression profiles [17]. The xenografts metastasize to lungs and lymph nodes (LNs) [17], but the role of hormones, if any, in tumor-cell dispersal is unclear. Lacking efficient solid tumor models, a recent study used systemically injected ER^+^PR^+^ MCF-7 cells to show that they can generate metastases in an E-dependent manner but that the initial homing and seeding steps with development of micrometastases do not require E [18]. Additionally, two dormancy models that included luminal cells were recently described [19],[20]. However, they do not address the role of hormones in metastasis or recurrence [4].

We previously demonstrated that in E-replete states, ER^+^PR^+^ orthotopic xenografts metastasize to distant LNs and occasionally to other organs [21]. Detailed immunohistochemistry (IHC) analyses of such tumors showed that during their expansion in mice, initially pure ER^+^PR^+^ cells develop cellular heterogeneity. At necropsy, presumptive “luminal” tumors contain at least one cell subpopulation we call “luminobasal” that is ER^−^PR^−^ and expresses cytokeratin 5 (CK5), a protein usually associated with basal-like cancers [11],[13],[22]. In clinical samples of luminal disease, similar basal-like, ER^−^PR^−^CK5^+^ cells, whose numbers increase with hormone therapies, can be found [23]. These hormone-resistant, possibly chemo-resistant cells are likely to have a poor prognosis. The heterogeneity raises questions about the identity of cell subpopulations in primary luminal disease that are responsible for metastatic engraftment and growth.

In this study, we develop a luminal metastases model and assess the role of E and P in metastatic engraftment and recurrence from dormancy. To short-circuit the cellular heterogeneity issue and study engraftment by each cell population independently, T47D-derived solid tumor xenografts were partitioned into their luminal (called E3) and luminobasal (called EWD8) subpopulations [22]. These, plus established luminal (MCF-7) and basal (MDA-MB-231) breast cancer cell lines, were tagged with luminescent and fluorescent markers for *in vivo* and *ex vivo* analyses. Cells were injected into the left ventricle of ovariectomized (ovx’d) immunocompromised mice, and their colonization and proliferation in distant organs was monitored in the absence of hormones or following E or E+P repletion. We found that luminobasal and basal cells generate metastases regardless of the hormonal state. In contrast, luminal cells rarely form metastases unless E or E+P is restored. The organs colonized by luminal and basal cells are similar and mimic the clinical pattern, dominated by bone. Despite initial injection of *in vitro* pure luminal cells, their metastases *in vivo* exhibit cellular heterogeneity, including outgrowth of ER^−^PR^−^CK5^+^ luminobasal subpopulations. These cells proliferate more slowly than surrounding luminal cells. Notably, although luminal cells seldom generate macrometastases in the absence of hormones, viable dormant micrometastases engraft at distant sites. If mice harboring such occult tumor cells are subsequently hormone supplemented, overt metastases materialize.

## Materials and methods

### Cell lines

MCF-7 human breast cancer cells were from Sam Brooks (Michigan Cancer Foundation); T47D cells were from Iafa Keydar (Tel Aviv University, Israel). The T47Dco subline described in Horwitz *et al.* [24] has low ER and exceptionally high PR that are not absolutely E-dependent. As described [22], E3 and EWD8 are sublines of T47Dco. The cell lines were derived from solid tumor xenografts grown in ovx’d NSG (non-obese diabetic/severe combined immunodeficient gamma) mice either without (E withdrawn, EWD) or with 17β-estradiol (E) supplementation. By gene profiling, E3 cells cluster with luminal cell lines and EWD8 cells cluster with basal-like triple-negative (TN) cell lines [22]. Their T47Dco origin was confirmed by short tandem repeat (STR) and karyotype analyses; their luminal versus basal-like classification and isogenicity were demonstrated by gene expression profiling [22]. BT-474 and MDA-MB-231 cells were from the ATCC (Manassas, VA, USA). These cells were also authenticated by STR analysis. All cells are mycoplasma-free.

To track metastases at necropsy, cells were tagged with ZsGreen (ZsG) by retroviral infection [21]. To quantify metastatic burden in living mice, cells were also modified to express luciferase. For this, pMSCV-Luciferase PGK-hygro (gift of Heide Ford, University of Colorado) was transfected into PT-67 packaging cells. ZsG-tagged cells were incubated in filtered, virus-containing supernatants and selected for hygromycin resistance. Luciferase expression was confirmed with a kit (Promega, Fitchburg, WI, USA). EWD8 cells were cultured in phenol red-free minimum essential medium (MEM) and 5% fetal bovine serum (FBS) depleted of hormones by incubation with dextran-coated charcoal pellets followed by filtration [22]. E3 cells were cultured in the same medium supplemented with 10 nM E. MCF-7 and MDA-MB-231 cells were grown as described [22].

### Intracardiac injections and metastases quantification

Animal protocols were approved by the University of Colorado Institutional Animal Care and Use Committee. Female NSG mice were ovx’d at 3 to 4 weeks (The Jackson Laboratory, Bar Harbor, ME, USA). Six-week-old mice were anesthetized with isoflurane and injected in the left ventricle with 10^5^ tumor cells suspended in 0.1 mL phosphate-buffered saline by using a 26G needle guided by ultrasound imaging (Vevo770; VisualSonics, Toronto, ON, Canada). Mice (10 or more per group) were implanted subcutaneously with silastic pellets containing 10 mg cellulose (C), 2 mg E + 8 mg cellulose (E), 2 mg C + 8 mg progesterone (P), or 2 mg E + 8 mg progesterone (E+P) that we previously showed release hormones at physiological levels for premenopausal women [11]. Body weights were recorded weekly. To switch from C to hormones after 8 weeks, mice were anesthetized and cellulose pellets were excised and replaced with C, E, P, or E+P-releasing pellets. Development and progression of metastases were quantified weekly by bioluminescent imaging with Xenogen *in vivo* imaging systems (IVIS) 200 (Caliper Life Sciences, Hopkinton, MA, USA) to capture photon flux, after anesthetized mice were intraperitoneally injected with 75 mg/kg D-Luciferin. Signal intensity was quantified with Living Image 2.60.1 software (Caliper Life Sciences). Mice were euthanized if imaging showed extensive metastases or at signs of morbidity. At necropsy, organs with ZsG fluorescent metastases were found by using UV light (Illumatool 9900; Lightools Research, Encinitas, CA, USA) coupled to a dissecting microscope (SZ-61; Olympus, Tokyo, Japan) and photographed with a digital camera (Olympus C-5050). Organs with ZsG fluorescence were collected and processed for IHC [21]. Additionally, apparently metastasis-free non-fluorescent organs, including lungs, liver, adrenals, LNs, and long bones, were harvested from mice in the C group.

### Three-dimensional colonies and histology

Briefly [25], for three-dimensional (3D) colonies, 4 × 10^4^ E3 or MCF-7 cells were plated into 8-well chambered slides (BD Biosciences, East Rutherford, NJ, USA) pre-coated with 60 μL growth factor-reduced phenol red-free Matrigel. Colonies were grown 1 week in phenol red-free MEM with 5% charcoal-stripped FBS, without hormones (C/ethanol, 1:1,000 vol/vol) or with E (10 nM), or E and P (100 nM), in media refreshed every 2 days. Matrigel blocks were embedded in Histogel (Richard-Allen Scientific, part of Thermo Fisher Scientific, Waltham, MA, USA), fixed overnight in 4% paraformaldehyde, switched to 70% (vol/vol) ethanol, and paraffin-embedded. Mouse organs were also fixed in 4% paraformaldehyde and processed as above. Bone samples were decalcified in 10% EDTA for 1 week prior to fixation. Paraffin-embedded samples were sectioned (4 μm) serially, deparaffinized in xylene, and rehydrated in graded alcohol. To locate micro- or macrometastases, every 5th or 10th section was stained with hematoxylin and eosin (H&E) until tumor cells appeared microscopically, after which continuous sections were saved for IHC. In some studies, CK8/18 IHC replaced H&E.

### Immunohistochemistry

For antigen retrieval, slides were heated in citrate buffer and sections were blocked 1 hour in 10% normal goat serum. Primary antibodies, including clones and concentrations or dilutions, are listed in Table S1 (Additional file 1). These were detected either with fluorescent secondary antibodies conjugated to AlexaFluor-488 or -555 (1:200, Invitrogen, Waltham, MA, USA) and counterstained with 4′,6-diamino-2-phenylindole (DAPI) or with horseradish peroxidase-labeled secondary antibodies and DAB+ chromogen/substrate (Dako, Glostrup, Denmark). Slides were imaged with a Nikon Eclipse E600 microscope (Nikon, Tokyo, Japan) equipped with a CoolSnap fx camera (Photometrics, Tucson, AZ, USA) operated by ImagePro software (Media Cybernetics, Rockville, MD, USA) [22]. For CK5, phosphor-histone H3 (pHH3) and nuclear proliferation marker (Ki67) quantification, photographs of at least five random 40× fields (3D colonies) or 100× fields (xenografts) per condition were used to calculate values.

### Clinical

Seventy-two primary tumors and matching metastases were collected from the archives of the Department of Pathology, Helsinki University Hospital, Finland, with approval from the ethics committee of the Helsinki University Central Hospital and with patient consent. Clinicopathological and other data have been published [26],[27]. Brain metastases from four patients were cut into 4-micron sections and stained by dual colorimetric IHC (Envision G/2 Doublestain; Dako) for PR (NeoMarkers, Freemont, CA, USA) and CK5 (Novocastra, Leica Biosystems, Nussloch, Germany) [22].

### Statistical analysis

Data are reported as mean ± standard error of the mean (SEM) of three or more independent experiments and were analyzed statistically with Prism v6.0 (GraphPad Software, La Jolla, CA, USA) by Student’s *t* test. Differences in survival curves among treatment groups were performed by using Kaplan-Meier semi-parametric method with the log-rank test. *P* values of less than 0.05 were considered statistically significant.

## Results

### Hormones promote metastasis of ER^+^PR^+^breast cancer cells

To analyze effects of ovarian hormones on metastases, ovx’d NSG mice implanted with control cellulose (C), E, or E+P-releasing pellets [11] were intracardiac (IC)-injected into the arterial circulation with 10^5^ tumor cells. Two luminal ER^+^PR^+^CK5^−^ (E3 and MCF-7) and two basal-like (EWD8 and MDA-MB-231) cell lines were compared. EWD8 are TN ER^−^PR^−^HER2^−^ but CK5^+^EGFR^+^ [22]; MDA-MB-231 are TN and CK5^−^EGFR^+^ [28]. Comparisons between E3 and EWD8 are especially useful since they are isogenic “twins”, having been derived from the same luminal parental cells [22]. All cells were modified to express luciferase and ZsG. To confirm cell subtype assignment prior to IC injections, the cells were grown as 3D colonies in phenol red-free Matrigel and analyzed for luminal and basal markers (Additional file 2). E3 cells are ER^+^PR^+^; MCF-7 cells are ER^+^PR^−^ in phenol red-free medium (but PR can be induced by E [29]). Both are CK5^−^ and vimentin^−^. EWD8 cells lack luminal markers and are CK5^+^vimentin^−^; MDA-MB-231 cells lack luminal markers and are CK5^−^vimentin^+^.

**Figure 1:**
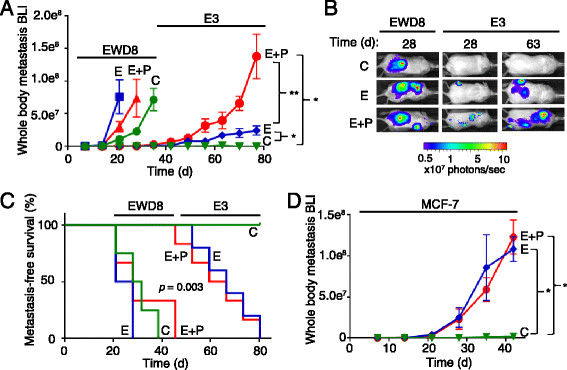
**Hormones are necessary for luminal tumor metastases. (A)** Ovariectomized (Ovx’d) non-obese diabetic/severe combined immunodeficient gamma (NSG) mice were intracardiac (IC)-injected with 10^5^ luciferase and ZsGreen-tagged basal-like estrogen withdrawn-line 8 (EWD8) or luminal E3 cells, and implanted with cellulose control (C) or with estrogen (E) or estrogen + progestin (E+P)-releasing pellets. Weekly quantitation used *in vivo* bioluminescent imaging (BLI). Data are presented as mean ± standard error of the mean (SEM) of BLI signal (n = 20 per treatment group). *P <0.05, **P <0.005, Student’s *t* test. **(B)** Representative luciferase BLI whole-body images. **(C)** Kaplan-Meier survival curves (n = 20 per treatment group); *P* = 0.003, log-rank test. **(D)** Ovx’d NSG mice were IC injected with 5 × 10^5^ luciferase and ZsGreen-tagged luminal MCF-7 cells and implanted with C, E, or E+P pellets. Weekly *in vivo* luciferase BLI quantitation. Data are presented as mean ± SEM of BLI signal (n = 20 per treatment group). *P <0.05, Student’s *t* test.

Progression of metastases in hormone-free or hormone-replete mice was quantified weekly by whole-body bioluminescent luciferase imaging (BLI). Basal-like ER^−^PR^−^ EWD8 cells were highly aggressive, producing extensive metastases within 20 days in the absence of hormones (C) (Figure 1A, B). Although E and E+P appear to accelerate EWD8 metastasis, the effects are not statistically significant (C versus E, *P* = 0.857; C versus E+P, *P* = 0.819) (Figure 1A). Mice had to be sacrificed after about 30 days. In contrast, mice injected with isogenic luminal ER^+^PR^+^CK5^−^ E3 cells appeared to be metastasis-free in the absence of hormones (C) up to the study termination of 80 days (Figure 1A). However, if E3-injected mice were supplemented with E or E+P starting on day 0, metastases began to develop after more than 28 days and the tumor burden increased weekly thereafter (Figure 1A, B). E-supplementation significantly (*P* <0.05) increased tumor burden relative to C, and addition of P to E exacerbated this (*P* <0.005).

As surrogates for overall health, mouse body weights were also monitored (Additional file 3A). Control (C) mice with EWD8 metastases lost weight rapidly, appeared sickly after 20 days, and were sacrificed early. C mice injected with E3 cells gained weight; ones receiving E or E+P maintained starting weights for up to 80 days. These data are reflected in metastasis-free survival as estimated by Kaplan-Meier plots (Figure 1C) with median survival of 30 days for EWD8 mice regardless of hormonal state, which is significantly (*P* = 0.003) less than the 65- to 70-day median survival for E3 mice on E or E+P, and the indefinite survival of control E3 mice.

To test a second luminal cell line, MCF-7 cells were IC injected into ovx’d mice without (C) or with E or E+P supplementation (Figure 1D). Like E3 cells, MCF-7 cells failed to produce metastases in the absence of hormones, but metastases were evident after 20 days in E- or E+P-supplemented mice. Compared with E3 cells, MCF-7 cells are more sensitive to E. In the presence of hormones, the MCF-7 metastatic burden increased progressively until the study was terminated at 45 days. Body weights remained relatively stable under all conditions (Additional file 3B). In sum, two different luminal breast cancer cell lines introduced into the circulation of immune compromised mice demonstrate that metastasis is promoted by ovarian hormones (Figure 1). In contrast, formation of metastases from basal-like cells is largely hormone-independent. Taken together, these data suggest that major effects of E or E+P are on the malignant luminal cells. This may be further modified by effects of hormones on the metastatic niche.

### Hormones promote luminal metastasis in multiple organs

**Figure 2:**
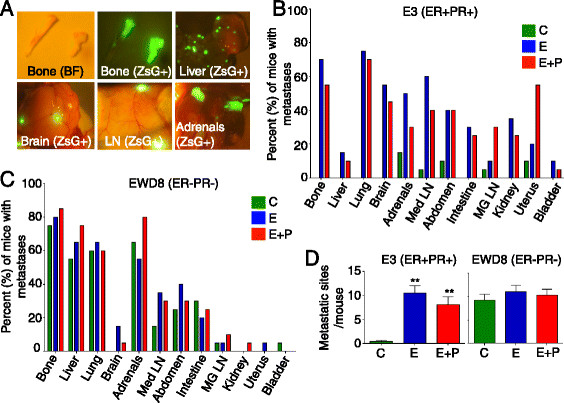
**Hormones control metastases of luminal but not basal-like tumor cells. (A)** Ovariectomized (Ovx’d) non-obese diabetic/severe combined immunodeficient gamma (NSG) mice were intracardiac-injected with 10^5^ luciferase and ZsGreen-tagged basal-like EWD8 or luminal E3 cells and implanted with cellulose (C) or with estrogen (E) or estrogen + progestin (E+P)-releasing pellets. Representative images at necropsy of ZsG fluorescent metastases in different organs are shown. BF, bright field image (n = 20 per treatment group). **(B)** Bar graph shows the percentage of mice in each treatment group—C (green), E (blue), and E+P (red)—with E3 metastases to distant organs. Data are presented as mean percentages per group (n = 20 per treatment group). **(C)** Bar graph shows the percentage of mice in each treatment group—C (green), E (blue), and E+P (red)—with EWD8 metastases to distant organs. Data are presented as mean percentages per group (n = 20 per treatment group). **(D)** Number of ZsG^+^ metastatic sites per mouse at necropsy for E3 and EWD8 cells under C (green), E (blue), or E+P (red) conditions. Data are presented as mean ± standard error of the mean (SEM) (n = 20 per treatment group). **P <0.005, Student’s *t* test.

Clinically, major sites of metastases are bones, liver, and lungs [5],[30]. ZsGreen fluorescence at necropsy (Figure 2A) was used to track the organ specificity of metastatic cells. In hormone-replete (but not hormone-deprived) mice, luminal E3 (Figure 2B) and MCF-7 cells (Additional file 4A) colonize multiple organs, including bones, liver, lungs, brain, adrenals, and LNs. Quantitation of the organ sites colonized indicate that E enhances the incidence of metastases (blue bars), which is not significantly altered by P (red bars) (Figure 2B and Additional file 4A). Overall, in the absence of hormones (green bars), the frequency of E3 and MCF-7 bone metastases in C mice is less than 2% but averages 65% in E- or E+P-replete mice. Luminal metastases to some organs (adrenals, for instance) exhibit some independence from exogenous hormones (Figure 2B and Additional file 4A) possibly because of endogenous steroid hormone biosynthesis [31]. Basal-like EWD8 (Figure 2C) or MDA-MB-231 cells (Additional file 4B) are highly aggressive and do not require hormones to form multi-organ metastases. In the case of bones, for example, basal cells formed metastases at 80% or greater frequency in both C and hormone-treated mice (Figure 2C and Additional file 4B).

Among the four cell lines, minor organ-specific differences were observed: Liver metastases were lower for E3 than MCF-7 cells, and abdominal/intestinal, mammary gland LN, renal, and uterine metastases were higher for E3 than MCF-7 cells (Figure 2B and Additional file 4A). Also, brain metastases were lower for EWD8 than for MDA-MB-231 cells (Figure 2C and Additional file 4B). Nonetheless, the number of metastatic sites observed for each cell line demonstrates the critical role of hormones (especially E) for metastases by luminal E3 and MCF-7 cells and the hormone independence for metastases by basal-like EWD8 and MDA-MB-231 cells.

### Cellular heterogeneity in luminal and basal metastases

**Figure 3:**
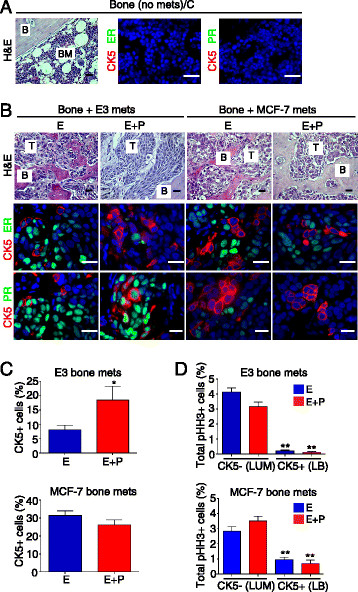
**Homogeneous ER** ^**+**^**PR** ^**+**^**CK5** ^**−**^**luminal E3 and MCF-7 cells generate heterogeneous bone metastases containing ER** ^**−**^**PR** ^**−**^**CK5** ^**+**^**luminobasal cells. (A)** Representative hematoxylin and eosin (H&E) and immunohistochemistry (IHC) of control (C) bones lacking metastases despite intracardiac (IC) injection of E3 or MCF-7 cells. H&E: (B) bone, (BM) marrow. Scale bars: 50 μm. IHC: estrogen receptor (ER) or progesterone receptor (PR) (green), cytokeratin 5 (CK5) (red), and 4′,6-diamino-2-phenylindole (DAPI) (blue) by immunofluorescent (IF) staining (n = 5 per group). Scale bars: 20 μm. **(B)** H&E and IHC of E- or E+P-treated mice with bones containing metastases after IC injection of E3 or MCF-7 cells. H&E: bone (B) and tumor (T) cells (n = 5 per group). Scale bars: 50 μm. IHC: ER or PR (green), CK5 (red) and DAPI (blue) by IF staining (n = 5 per group). Scale bars: 20 μm. **(C)** Percentage of CK5^+^ cells in E- or E+P-treated E3 and MCF-7 bone metastases. Mean ± standard error of the mean (SEM) values are shown (n = 5 per group). *P <0.05, Student’s *t* test. **(D)** Proliferation rates of CK5^+^ and CK5^−^ cells measured by phosphor-histone H3 (pHH3) in E- and E+P-treated E3 and MCF-7 bone metastases. Mean ± SEM values are shown (n = 5 per group). **P <0.005, Student’s *t* test.

Over 70% of patients with breast cancer succumb to lytic bone lesions that cause fractures, pain, and associated complications [32]. Since clinically, metastases can contain cells that differ from those at the primary site [33], experimental bones were sectioned and analyzed by H&E and IHC. Multiple bone sections of ovx’d C mice injected with luminal E3 or MCF-7 yielded no tumor cells (Figure 3A) confirming the necropsy data. However, more than 50% of bones in E- or E+P-supplemented mice contained tumor cells (Figure 3B). These were generally found in long bones and spine, and were characterized by islands of malignant cells infiltrating the marrow space, associated with variable bone destruction.

Unlike the homogeneous ER^+^PR^+^CK5^−^ luminal state of E3 and MCF-7 cells at the time of injection (Additional file 2), bone metastases are heterogeneous (Figure 3B, middle and lower panels), consisting not only of the expected ER^+^PR^+^ luminal cells (green nuclei) but also of multiple basal-like ER^−^PR^−^CK5^+^ luminobasal cells (red cytoplasm) [22]. E3 metastases contained about 8% luminobasal cells in the presence of E, which were significantly (*P* = 0.037) increased to about 18% by addition of P to E (Figure 3C, top panel). MCF-7 metastases contained high levels of luminobasal cells (25% to 30%) in both E and E+P (Figure 3C, bottom panel).

Although hormone-dependent acquisition of heterogeneity is not observed for cells grown on plastic, it can be studied in 3D colony assays (Additional file 5). For instance, E3 colonies contain 0% CK5^+^ cells in E and 14% CK5^+^ cells in E+P. MCF-7 colonies contain about 3% CK5^+^ cells in E or E+P. Differences between colony assays and *in vivo* metastases may be due to signals contributed by the metastatic niche.

Detailed marker analyses of luminal E3 and MCF-7 bone metastases are shown in Additional file 6A and B. The ER^−^PR^−^ subpopulation loses the luminal markers CK8/18 and claudin 3 (CLD3) and is HER2^−^ (unlike BT-474 controls). Luminal bone metastases also lack the mesenchymal marker vimentin, unlike basal-like MDA-MB-231 metastases that retain vimentin (Additional file 6A and B).

Basal-like EWD8 and MDA-MB-231 cells also colonize bone but do so even in the absence of hormones (Additional file 7A). Histopathology again shows malignant epithelial cells infiltrating the marrow with bone destruction; EWD8 infiltrates also show signs of desmoplasia (Additional file 7A, top H&E panel). Receptor conversion in clinical cases has been reported [34],[35]. Because EWD8 cells have luminal parentage [22], we looked for reversions by using ER and PR as markers. However, EWD8 metastases uniformly retain the basal marker CK5 and are ER^−^, PR^−^, and vimentin^−^ (Additional file 7A). A “double negative” ER^−^PR^−^CK5^−^ subpopulation was also detected (Additional file 7A). Double-negative cells resemble MDA-MB-231 metastases with regard to CK5 loss, but unlike MDA-231 cells, they lack vimentin (Additional file 7A). These analyses demonstrate interesting cellular heterogeneity even within basal-like metastases.

In previous studies of primary luminal cancers, we showed that the ER^+^PR^+^ population suppresses mitosis of the ER^−^PR^−^ subpopulation [23]. The latter would therefore be both hormone-resistant because of a lack of receptors and chemotherapy-resistant because of quiescence [23]. To assess proliferation of cell subpopulations in our models, sections of E3, MCF-7, and EWD8 bone metastases from E- or E+P-treated mice were dual-stained for CK5 and the mitosis marker pHH3 [36]. We find that regardless of the hormone state, in both E3 and MCF-7 bone metastases (Figure 3D), the ER^−^PR^−^CK5^+^ luminobasal (LB) subpopulation is significantly (*P* <0.005) growth-suppressed compared with its ER^+^PR^+^CK5^−^ luminal (LUM) neighbors. In EWD8 bone metastases (Additional file 7B), growth of the pre-existing CK5^+^ population is significantly (*P* <0.005) reduced compared with the newly formed double-negative ER^−^PR^−^CK5^−^ subpopulation. In sum, in addition to marker heterogeneity, cell growth is heterogeneous in metastases, making therapy difficult.

Additional file 8A shows brain metastases caused by E3 and MCF-7 cells. Like bone, brain metastases are heterogeneous, containing both the injected ER^+^PR^+^CK5^−^ luminal population (green) and the newly arisen ER^−^PR^−^CK5^+^ luminobasal subpopulation (red). Therefore, this phenotypic conversion is not organ-specific. Similar cellular heterogeneity marks luminal metastases of patients as shown in a clinical sample of brain metastases (Additional file 8B) [27]. Overall, these data demonstrate that injection of apparently homogeneous tumor cells generates metastases with heterogeneous cell populations, partly in response to hormones. Furthermore, these subpopulations can exhibit divergent proliferation rates.

### Luminal tumor dormancy and reactivation by hormones

**Figure 4:**
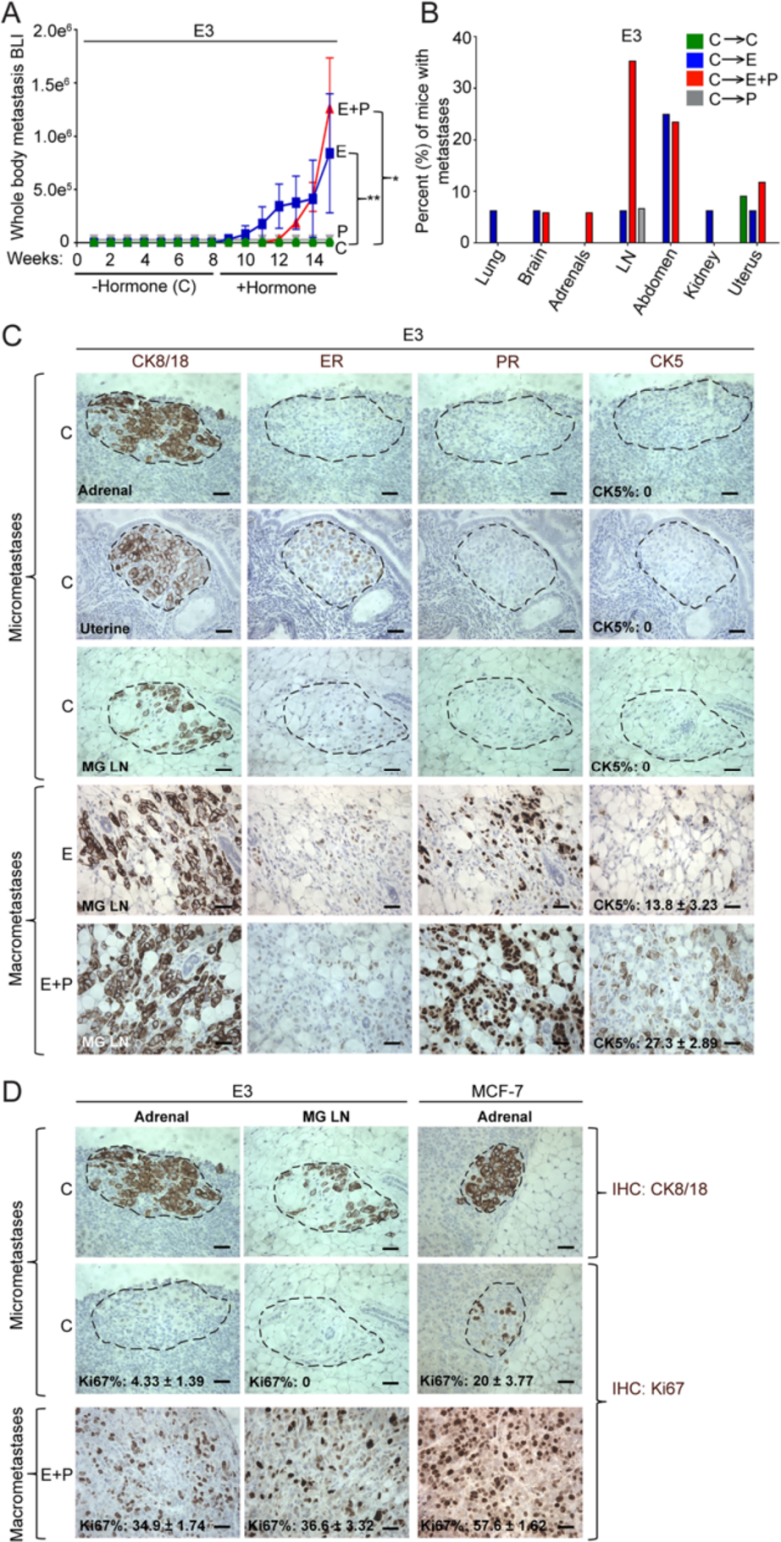
**Hormones reactivate dormant luminal micrometastases. (A)** Ovariectomized (Ovx’d) non-obese diabetic/severe combined immunodeficient gamma (NSG) mice were intracardiac (IC)-injected with 10^5^ luciferase- and ZsG-tagged E3 cells without hormone supplementation (cellulose, or C) and monitored for 8 weeks. At week 8, the C pellet was removed and replaced with C (n = 11), estrogen (E) (n = 16), progestin (P) (n = 15), or estrogen + progestin (E+P) (n = 17)-releasing pellets for another 7 to 8 weeks. Weekly bioluminescent luciferase imaging (BLI) imaging in live mice by *in vivo* imaging systems (IVIS). Data are presented as mean ± standard error of the mean (SEM) of BLI signal. *P <0.05, **P <0.005, Student’s *t* test. **(B)** Bar graph shows the percentage of mice in each treatment group—C (green), E (blue), E+P (red), and P (gray)—with E3 metastases in distant organs without (C) or with E, P, or E+P restoration. Data are presented as mean percentages per group. **(C)** Immunohistochemistry (IHC) for CK8/18, ER, PR, and CK5 in organs with E3 micrometastases (C) and macrometastases (E or E+P). For percentage of CK5^+^ cells, mean ± SEM values are shown (n = 3 per group). Scale bars: 50 μm. **(D)** IHC for CK8/18 and nuclear proliferation marker (Ki67) in organs with E3 or MCF-7 micrometastases (C) and macrometastases (E+P). For percentage of Ki67^+^ cells, mean ± SEM values are shown (n = 3 per group). Scale bars: 50 μm.

Our models demonstrate that IC-injected malignant luminal cells form macrometastases in hormone-supplemented but not in hormone-free ovx’d mice. What happened to the disseminated tumor cells injected into hormone-free mice? To address this, hormone-free ovx’d C mice were IC-injected with 10^5^ Luc/ZsG-E3 or Luc/ZsG-MCF-7 cells and monitored for 8 weeks by IVIS (Figure 4A). Few metastases were imaged by IVIS despite the fact that 8 weeks is more than sufficient for these to be visible in hormone-supplemented mice (Figure 1A, 1D). At 8 weeks, the C pellets were removed and replaced with C, E, P, or E+P-releasing pellets, and mice were monitored for another 8 weeks. As shown for E3 injected mice (Figure 4A), under C or P-restored conditions, mice remained metastases-free. However, metastases emerged in E- or E+P-restored mice. Results from IVIS imaging were confirmed at necropsy (Figure 4B), with few E3 ZsG^+^ fluorescent deposits after 16 weeks in organs of C (1/11; 9%) or P-treated mice (1/15; 6%) but robust ZsG+ metastases in one or more organs of E (8/16; 50%) or E+P (10/17; about 59%) mice. Guided by ZsG fluorescence, organs were sectioned and stained with H&E. This confirmed the presence of macrometastases in multiple organs of E- or E+P-replete mice, most commonly in lungs, brain, adrenals, LNs, abdomen, kidney, and uterus (Figure 4B). (Organs with macrometastases often had micrometastases in adjacent regions.) C or P mice showed few if any metastases in random H&E-stained sections of similar organs (Figure 4B).

To explain how luminal metastases could arise in C mice that appeared to be metastases-free prior to hormone resumption, we reasoned that one or more organs must have harbored micrometastases that had escaped detection because of random sectioning or fluorescence below the limits of detection. We therefore returned to E3-injected C mice necropsied at 16 weeks, and systematically serial-sectioned select entire organs, immunostaining every 10th 4 μm section for the luminal marker CK8/18. This marker detects micrometastases—defined as fewer than 100 cells—more effectively than H&E. We found CK8/18^+^ micrometastases in adrenal glands, uteri, and mammary gland LNs of C mice (Figure 4C). For comparison, CK8/18^+^ E3 macrometastases in mammary gland LNs of E- and E+P-treated mice are also shown (Figure 4C). Apparently metastases-free C mice injected with MCF-7 cells also harbored CK8/18^+^ micrometastases in adrenal glands, which are compared with CK8/18^+^ MCF-7 macrometastases in adrenal glands of E- and E+P-treated mice (Additional file 9A).

To contrast molecular markers of micro- versus macrometastases, sections adjacent to CK8/18^+^ ones were immunostained for ER, PR, and CK5. Control E3 micrometastases were surprisingly PR^−^ and also CK5^−^ (Figure 4C). MCF-7 micrometastases were PR^−^ with about 9% CK5^+^ cells (Additional file 9A). E3 macrometastases, derived from hormone-treated mice, were ER^+^, strongly PR^+^ with about 14 (E) or 27% (E+P) CK5^+^ cells (Figure 4C). Similarly, E- or E+P-treated MCF-7 macrometastases were ER^+^, PR^+^, and CK5^+^ (Additional file 9A). Of note, PR^+^ and CK5^+^ heterogeneity are restored or enhanced in dormant tumors reactivated by hormones in both E3 and MCF-7 adrenal and LN metastases (Additional file 9B). As described for bone metastases (Figure 3D), the CK5^+^ luminobasal subpopulation is markedly growth-suppressed compared with its CK5^−^ neighbors (Additional file 9C).

To contrast proliferation rates between macro- and micrometastases, Ki67 was quantified (Figure 4D); 35% to 37% of adrenal or mammary gland LN E+P-treated E3 macrometastatic cells were proliferating, compared with 0% to 4% of similar micrometastatic cells (Figure 4D). Thus, E3 micrometastases are functionally dormant as defined clinically [37],[38]. MCF-7 cells are more proliferative even as micrometastases with about 20% Ki67^+^ cells (Figure 4D), which may explain their more aggressive behavior (compare Figure 1A with Figure 1D).

Overall, these data demonstrate that, even in the absence of hormones, luminal tumor cells disseminated through the circulation can, and do, implant and survive at metastatic sites; that entry into “dormancy” varies among tumors (as it does here for T47D-derived versus MCF-7 cells) making some less perilous than others; and that some cells enter a state of proliferative stasis from which they can escape if E or E+P is restored. The clinical implications are grave with regard to hormone supplementation for women who are breast cancer survivors or have occult disease.

## Discussion

### Models

Though considered a stringent “intrinsic” molecular subtype definable by presence of steroid receptors, luminal breast cancer is a complex disease. Among other variables, tumors that are ER^+^PR^+^, ER^+^PR^−^ or ER^−^PR^+^, HER2^−^ or HER2^+^, or low- or high-grade are all classified as luminal [39],[40]. Tumors that may have initiated years or decades earlier are diagnosed in the pre-, peri-, and post-menopausal years, each with the attendant hormonal changes that differ from woman to woman. Until recently, clinical assays, including gene expression profiling, assessed the bulk of a breast cancer to assign its subtype. However, such assays cannot define intratumoral cell heterogeneity [41]. IHC analyses clearly demonstrate that invariably, individual luminal tumors consist of cell mixtures, only one of which is the classic ER^+^PR^+^ hormone-responsive cell. Here, we use IHC to examine the role of ovarian hormones in luminal breast cancer metastasis by using four experimental cells: two luminal and two basal. Like their clinical counterparts, the models are variable: MCF-7 are ER^+^ but need estradiol to induce PR; T47Dco and their E3 derivatives are ER^+^ with partially constitutive elevated PR. Both are CK5^−^. EWD8, also derived from T47Dco, is ER^−^PR^−^ and CK5^+^; MDA-MB-231 is basal-like and CK5^−^. We describe a role for hormones in development of luminal metastases, the cellular heterogeneity of micro- and macrometastases arising from apparently homogeneous cell models, a PR^−^ dormant state that may be a sanctuary from therapies, and a role for hormones in escape from dormancy. To the best of our knowledge, these observations are novel.

The cellular heterogeneity is clearly demonstrable with just two markers—PR and CK5—that theoretically generate four possible cell types: luminal *PR* ^*+*^*CK5* ^*−*^, luminobasal *PR* ^*−*^*CK5* ^*+*^, double-negative *PR* ^*−*^*CK5* ^*−*^, and double-positive PR^+^CK5^+^. To various degrees, we observe all four in clinical and model luminal disease. It is not unreasonable to postulate that, if more markers were analyzed, even greater heterogeneity would be observed, ultimately demonstrating that every single luminal tumor is unique in its cellular composition and therapy vulnerability. This has clear implications for disease management.

### Hormones promote metastasis of ER^+^PR^+^cells

As has been elegantly demonstrated, metastasis requires completion of a complex series of steps, including escape of tumor cells from the primary site into surrounding tissues, their survival and transport via lymphatics and blood vessels, and their capture and expansion in distant organs [33]. We previously showed that xenografted solid luminal tumors efficiently metastasize to local and distant LNs [21]. On the other hand, compared with basal tumors, luminal tumor metastasis to distant organs is highly inefficient, requiring months of observation [42] and risking premature mouse attrition. Xenograft tumor volumes that are achieved in 20 to 30 days with basal cells require 150 to 200 days with luminal cells. Therefore, to design studies in which hormone supplementation is the major variable and in which different cell subpopulations can be analyzed, we used IC injection, which circumvents the first steps in the metastasis cascade. Under these conditions, basal metastases were quantifiable in 20 to 30 days; luminal metastases in 40 to 80 days (Figure 1). Parenthetically, these numbers suggest that a major impediment to experimental studies of luminal metastases from solid tumors is the early local invasion and intravasation steps [33] but that once tumor cells are in the blood, later events unfold rapidly regardless of tumor subtype.

Based on our experience and that of others [43], these early steps cannot be hastened by hormones. On the other hand, the present studies show for the first time that for receptor-positive luminal cells, the later steps are hormone-dependent. Compared with basal-like MDA-MB-231 and EWD8 cells, which metastasize efficiently in ovx’d mice with or without hormones, luminal E3 and MCF-7 cells do so mainly if hormones are restored. Estrogens appear to be key. Effects of P are variable, amplifying the E-dependent metastasis rate of E3 cells (Figure 1A) but failing to do so for MCF-7 cells (Figure 1D). This is another example of luminal tumor variability. These data validate the clinical practice of using adjuvant therapies that suppress E production or ER signaling to slow development of luminal metastases [7]. However, such preventive strategies would fail for the ER^−^PR^−^CK5^+^ luminobasal subpopulations commonly found in luminal disease [22],[23]. This may explain why, compared with ER^+^PR^+^ primary tumors, ER tumors are lost in about 25% of metastases and PR tumors are lost at an even greater rate [44],[45] and suggests that adjuvant combination therapies that target both cell types might be extremely effective. However, chemotherapies are unlikely to work since luminobasal cells are quiescent when mixed with luminal cells (Figure 3D and Additional file 9C) [23].

### Hormones promote luminal metastases at multiple organs

Luminal and basal cells tend to colonize the same organs: bones, liver, lungs, brain, and adrenals (Figure 2 and Additional file 4). The same sites are colonized in patients regardless of breast tumor subtype [46]. However, this tends to be hormone-dependent for ER^+^PR^+^ luminal cells and hormone-independent for ER^−^PR^−^ basal cells, suggesting that the exogenous hormones target the malignant cells directly. Of course, the metastatic niche may also be targeted by hormones [47]. Additionally, some organs, like adrenals and brain and tissues like fat, are capable of endogenous E and P biosynthesis [31]. Such extragonadal hormone production increases with age [48], which might drive the slow but eventual development of experimental luminal metastases from solid tumor xenografts in rodents or hormone-dependent metastases in postmenopausal women. Significantly, in women, no life stage is entirely depleted of ovarian hormones.

### Heterogeneity of metastases from pure luminal progenitors

The origins of intratumoral heterogeneity remain under intense debate. Explanations include differentiation from cancer stem cells, clonal expansion of intrinsic subpopulations, microenvironmental selection, random mutations creating genetic diversity, and epigenetic switches [33],[41]. It is likely that at one time or other all of these come into play. The present data focus on bone metastases, although heterogeneity is observed in other sites as well (Additional file 8; data not shown). In our studies, despite having injected apparently homogeneous luminal cells (Additional file 2), their metastases are heterogeneous (Figure 3 and Additional file 6). E3 metastases contain about 8% ER^−^PR^−^ luminobasal cells in E-supplemented mice and about 18% luminobasal cells in E+P-supplemented mice (Figure 3C) consistent with the fact that P induces luminobasal cells [11]. Remarkably, although the injected parental MCF-7 cells appear to be uniformly CK5^−^, there is considerable intratumoral heterogeneity in their metastases with 25% to 30% of cells ER^−^PR^−^CK5^+^ in E or E+P conditions (Figure 3C). Since the injected cells were homogeneous (Additional file 2), the heterogeneity had to arise within the metastatic niche and hormones play a role in inducing heterogeneity (Additional file 5). However, we cannot rule out that rare non-luminal rogue cell variants or stem cells were contained in the parental cells at the time of injection. It is also difficult at present to dissect out whether hormones generate the diversity (versus simply being growth-permissive for some but not all subpopulations).

### Dormant luminal tumors: sheltered from therapies and activated by hormones

Tumor dormancy is well documented with luminal disease having a propensity to recur even decades after first diagnosis [37],[49],[50]. Anatomical compartments in which minimal residual disease have been detected include bone marrow, LNs, and blood [49], where, it is postulated, the tumor mass remains microscopic by evading immune surveillance or failing to recruit a vascular bed [49],[50]. Although there are limitations to the methods we use to detect micrometastases, we find dormant luminal micrometastases in some organs of control ovx’d mice. Micrometastases appear to have a different molecular composition than their macromolecular counterparts. Interestingly, both E3 and MCF-7 micrometastases are ER^+^PR^−^ (Figure 4C and Additional file 9A). This could have serious consequences since loss of PR is a poor prognostic factor in ER^+^ breast cancers and plays a role in tamoxifen resistance [51]–[53]. We postulate that PR negativity, coupled with intrinsic mitotic quiescence of the CK5^+^ subpopulation, shelters dormant luminal micrometastases from most current therapies.

We also show a role for women’s hormones in luminal-tumor arousal from dormancy (Figure 4). Of interest is the fact that all proposed dormancy mechanisms focus on the metastatic site (rather than the primary site), making our models particularly relevant [37],[49]. Since NSG mice are immunodeficient, emergence from dormancy after hormone replacement is unlikely to involve immune mechanisms [49]. On the other hand, angiogenesis could play a role [37],[49]. Either cells in the metastatic niche or the ER^+^ tumor cells residing therein could be targets of hormones that, among other things, upregulate hypoxia inducible factor-1, VEGF, and other factors involved in vasculogenesis [47],[54],[55]. In the latter scenario, the hormone-activated luminal tumor cells would be direct participants in expanding the vasculature that supports their growth.

## Conclusions

We compare luminal and basal-like breast cancer cells for their propensity to develop metastases or undergo dormancy prior to resuming metastatic growth; show that for luminal cells, both processes are hormone-dependent; and suggest that dormant luminal cells may be sheltered from therapies and that, compared with the injected cells, their metastases contain variant cell subpopulations. Translated to the clinic, these data explain, on the one hand, the value of adjuvant E-suppressive therapies and even chemotherapies to delay metastases but perhaps their failure eventually to prevent metastases from previously disseminated cells. The data also point to the danger of initiating hormone replacement therapies in breast cancer survivors who may harbor occult, dormant micrometastases. Furthermore, about 9% of women who have never had a diagnosis of breast cancer have undiagnosed occult invasive disease or ductal carcinoma *in situ* at the time of death from other causes [56]. This microdisease reservoir also could be inadvertently activated by hormones [15].

## Additional files

## Electronic supplementary material

Additional file 1: List of reagents and antibodies. (PDF 320 KB)

Additional file 2: **Immunohistochemistry (IHC) of three-dimensional (3D) colonies formed from luminal estrogen receptor-positive/progesterone receptor-positive (ER** ^**+**^**PR** ^**+**^**) and basal-like ER** ^**−**^**PR** ^**−**^**cell lines prior to intracardiac (IC) injections.** E3, MCF-7, estrogen withdrawn-line 8 (EWD8), and MDA-MB-231 cells were grown as 3D Matrigel colonies and processed for IHC. Sections underwent dual immunofluorescent staining for cytokeratin 5 (CK5) (red), ER, PR, or vimentin (green) and were counterstained with 4′,6-diamino-2-phenylindole (DAPI) (blue). Images are representative of three independent cultures. Scale bars: 20 μm. (PDF 2 MB)

Additional file 3: **Body weights of mice during observation for development of metastases (A).** Total body weights were measured weekly for ovariectomized (ovx’d) mice intracardiac (IC)-injected with estrogen withdrawn-line 8 (EWD8) or E3 cells treated with control (C), estrogen (E), or estrogen + progestin (E+P) for 80 days. Data are presented as mean ± standard error of the mean (SEM) (n = 20 per group). **(B)** Total body weights were measured weekly for ovx’d mice IC-injected with MCF-7 cells treated with C, E, or E+P for about 40 days. Data are presented as mean ± SEM (n = 20 per group). (PDF 218 KB)

Additional file 4: **Hormonal regulation of MCF-7 and MDA-231 cell metastases.** **(A)** Bar graph shows the percentage of mice in each treatment group—control (C) (green), estrogen (E) (blue), and estrogen + progestin (E+P) (red)—with MCF-7 metastases to distant organs. Data are presented as mean percentages per group (n = 20 per treatment group). **(B)** Bar graph shows the percentage of mice in each treatment group—C (green), E (blue), and E+P (red)—with MDA-231 metastases to distant organs. Data are presented as mean percentages per group (n = 10 per treatment group). **(C)** Number of ZsGreen-positive (ZsG^+^) metastatic sites per mouse intracardiac (IC)-injected with MCF-7 or MDA-MB-231 cells in C, E, or E+P states. Data are presented as mean ± standard error of the mean (SEM) (n = 20 per group for MCF-7; n = 10 per group for MDA-231). **P <0.005, Student’s *t* test. (PDF 239 KB)

Additional file 5: **Hormones increase the number of cytokeratin 5-positive (CK5** ^**+**^**) cells in three-dimensional (3D) luminal colonies.** **(A)** E3 cells were grown as 3D colonies in phenol red-free growth factor-reduced Matrigel and treated with control (ethanol, 1:1,000 vol/vol), 10 nM estrogen (E), or 100 nM progestin (P) for 1 week. Percentages of CK5^+^ cells are presented as mean ± standard error of the mean (SEM) values. Scale bars: 50 μm. **(B)** MCF-7 cells were grown as 3D colonies in phenol red-free growth factor-reduced Matrigel and treated with control, 10 nM E, and 100 nM P for 1 week. Percentages of CK5^+^ cells are presented as mean ± SEM values. Scale bars: 50 μm. Both images are representative of three independent experiments. (PDF 3 MB)

Additional file 6: **Immunohistochemistry (IHC) of luminal E3 or MCF-7 bone metastases showing heterogeneity for receptors and cytokeratin 5 (CK5), plus other markers.** **(A)** Ovariectomized (Ovx’d) mice were intracardiac (IC)-injected with E3 cells and treated with control (C), estrogen (E), or estrogen + progestin (E+P). IHC: Bone sections were stained for CK8/18, CLD3, or HER2 (green); CK5 (red); and 4′,6-diamino-2-phenylindole (DAPI) counterstain (blue). Representative images are shown (n = 4 per group). BT474 and MDA-MB-231 cells were used as positive controls for HER2 or vimentin. Scale bars: 20 μm. **(B)** Ovx’d mice were IC-injected with MCF-7 cells and treated with C, E, or E+P. IHC: Bone sections were stained for CK8/18 or HER2 (green), CK5 (red), and DAPI (blue). Representative images are shown (n = 3 per group). BT474 and MDA-MB-231 cells were used as positive controls. Scale bars: 20 μm. (PDF 9 MB)

Additional file 7: **Heterogeneity of marker expression in basal-like estrogen withdrawn-line 8 (EWD8) or MDA-231 bone metastases.** **(A)** Representative hematoxylin and eosin (H&E) and immunohistochemistry (IHC) of EWD8 or MDA-MB-231 bone metastases in mice treated with control (C), estrogen (E), or estrogen + progestin (E+P). H&E: bone (B), tumor (T). Scale bars: 50 μm. IHC: Dual staining for ER, PR, or vimentin (green), CK5 (red), and 4′,6-diamino-2-phenylindole (DAPI) (blue) is indicated (n = 5 per group). Scale bars: 20 μm. **(B)** Proliferation rate of luminobasal (LB) ER^+^PR^+^CK5^+^ and double-negative (DN) ER^−^PR^−^CK5^−^ subpopulations measured with phosphor-histone H3 (pHH3) in C, E, and E+P-treated EWD8 bone metastases. Mean ± standard error of the mean (SEM) values are shown (n = 4 per group). **P <0.005, Student’s *t* test. (PDF 7 MB)

Additional file 8: **Heterogeneity of luminal brain metastases.** **(A)** Hematoxylin and eosin (H&E) and immunohistochemistry (IHC) of E3 and MCF-7 brain metastases in mice treated with control (C), estrogen (E), or estrogen + progestin (E+P). H&E: Tumor-free or normal brain (N), tumor cells (T). Scale bars: 50 μm. IHC: Dual staining for progesterone receptor (PR) or cytokeratin 8/18 (CK8/18) (green), CK5 (red), and 4′,6-diamino-2-phenylindole (DAPI) (blue) (n = 3 per group). Scale bars: 20 μm. **(B)** Paraffin sections of brain metastases from a patient with luminal breast cancer; dual colorimetric staining for CK5 (pink) and PR (brown). Scale bars: 20 μm. (PDF 8 MB)

Additional file 9: **Hormones increase progesterone receptor (PR) and cytokeratin 5 (CK5) expression in MCF-7 macrometastases, but CK5** ^**+**^**cells are relatively quiescent.** **(A)** Immunohistochemistry (IHC) for CK8/18, estrogen receptor (ER), PR, and CK5 in MCF-7 adrenal gland micrometastases formed in the absence of hormones (C), versus macrometastases formed with estrogen (E) and estrogen + progestin (E+P). Percentages of CK5^+^ cells are presented as mean ± standard error of the mean (SEM) values (n = 3 per group). Scale bars: 50 μm. **(B)** IHC for ER or PR (green), CK5 (red), and 4′,6-diamino-2-phenylindole (DAPI) (blue) of E3 and MCF-7 macrometastases in adrenals or LNs (n = 5 per group). Scale bars: 20 μm. **(C)** Proliferation rates of CK5^+^ and CK5^−^ cells measured by phosphor-histone H3-positive (pHH3^+^) in E+P-treated E3 or MCF-7 adrenal (adr.) gland and LN metastases aroused from dormancy. Mean ± SEM values are presented (n = 3 per group). **P <0.005, Student’s *t* test. (PDF 3 MB)

Below are the links to the authors’ original submitted files for images.

Authors’ original file for figure 1

Authors’ original file for figure 2

Authors’ original file for figure 3

Authors’ original file for figure 4

## Abbreviations

3D: three-dimensional
C: cellulose
CK: cytokeratin
E: estrogen
E3: estrogen-derived line 3
ER: estrogen receptor
EWD8: estrogen withdrawn-line 8
FBS: fetal bovine serum
H&E: hematoxylin and eosin
HRT: hormone replacement therapy
IC: intracardiac
IHC: immunohistochemistry
IVIS: *in vivo* imaging systems
Ki67: nuclear proliferation marker
LN: lymph node
MEM: minimum essential medium
NSG: non-obese diabetic/severe combined immunodeficient gamma
ovx’d: ovariectomized
P: progesterone
pHH3: phospho-histone H3
PR: progesterone receptor
STR: short tandem repeat
TN: triple-negative
WHI: Women’s Health Initiative
ZsG: ZsGreen
