# Supplementary material for: Luminal breast cancer metastases and tumor arousal from dormancy are promoted by direct actions of estradiol and progesterone on the malignant cells
Source: Breast Cancer Res. 2014 Dec 5;16:489. doi: 10.1186/s13058-014-0489-4 (PMC4303198; doi:10.1186/s13058-014-0489-4)
Supplement: Supplementary file 1 — Additional file 1: List of reagents and antibodies. (PDF 320 KB) [file 13058_2014_489_MOESM1_ESM.pdf]

Table S1. Reagents and Antibodies

Chemical/Antibody (ID)	Target/Application	Dilution/Concentration (w/v)	Source
17β-estradiol	ER	10 nM	Sigma
Progesterone	PR	100 nM	Sigma
CK5 (NCL-L-CK5)	IHC, IF	1:100 (0.5μg/ml)	Leica Microsystems
CK5 (2290-1)	IHC, IF	1:200 (0.05μg/ml)	Epitomics
CK8/18 (NCL-L-503)	IHC, IF	1:100 (4.2μg/ml)	Leica Microsystems
Claudin 3 (ab15102)	IF	1:100 (2μg/ml)	Abcam
EGFR (06847)	IF, IHC	1:400 (2.5μg/ml)	Millipore
ER-α (SP1)	IHC, IF	1:100*	Neomarkers
Her2 (SP3)	IF	1:50*	Neomarkers
Ki67 (MIB-1)	IHC	1:50*	DAKO
PR (Clone 1294)	IHC, IF	1:100 (0.266 μg/ml)	DAKO
PR (SP2)	IHC, IF	1:200*	Neomarkers
Phospho Histone H3 (Ser10)	IF	1:200 (1μg/ml)	Millipore
Vimentin (V9)	IF	1:100 (2μg/ml)	Santa Cruz Biotechnology

Abbreviations: IHC, immunohistochemistry; IF, immunofluorescence

**ER, HER2, Ki67 and PR antibodies indicated were supplied as tissue culture supernatants*
