# Supplementary figures and images for: Luminal breast cancer metastases and tumor arousal from dormancy are promoted by direct actions of estradiol and progesterone on the malignant cells
Source: Breast Cancer Res. 2014 Dec 5;16:489. doi: 10.1186/s13058-014-0489-4 (PMC4303198; doi:10.1186/s13058-014-0489-4)

Additional File 2

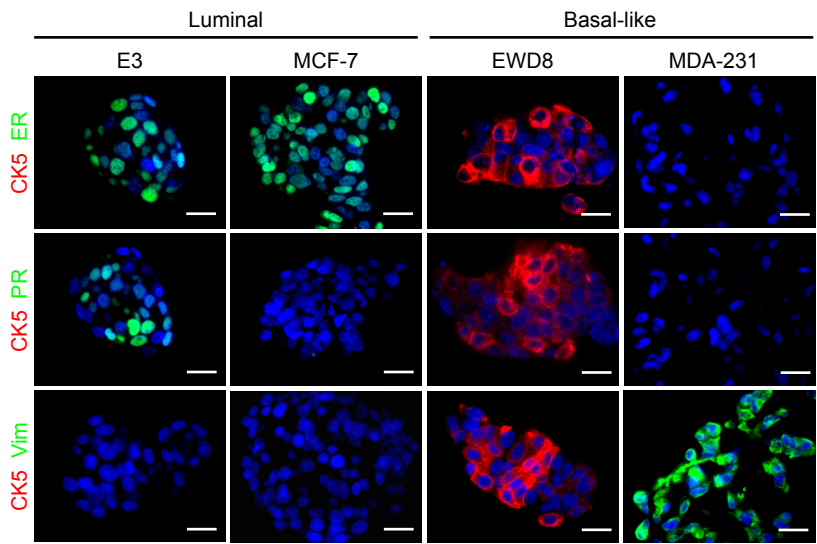

Supplement: Supplementary file 2 — Additional file 2: Immunohistochemistry (IHC) of three-dimensional (3D) colonies formed from luminal estrogen receptor-positive/progesterone receptor-positive (ER +PR +) and basal-like ER −PR −cell lines prior to intracardiac (IC) injections. E3, MCF-7, estrogen withdrawn-line 8 (EWD8), and MDA-MB-231 cells were grown as 3D Matrigel colonies and processed for IHC. Sections underwent dual immunofluorescent staining for cytokeratin 5 (CK5) (red), ER, PR, or vimentin (green) and were counterstained with 4′,6-diamino-2-phenylindole (DAPI) (blue). Images are representative of three independent cultures. Scale bars: 20 μm. (PDF 2 MB) [file 13058_2014_489_MOESM2_ESM.pdf]

### Additional File 3

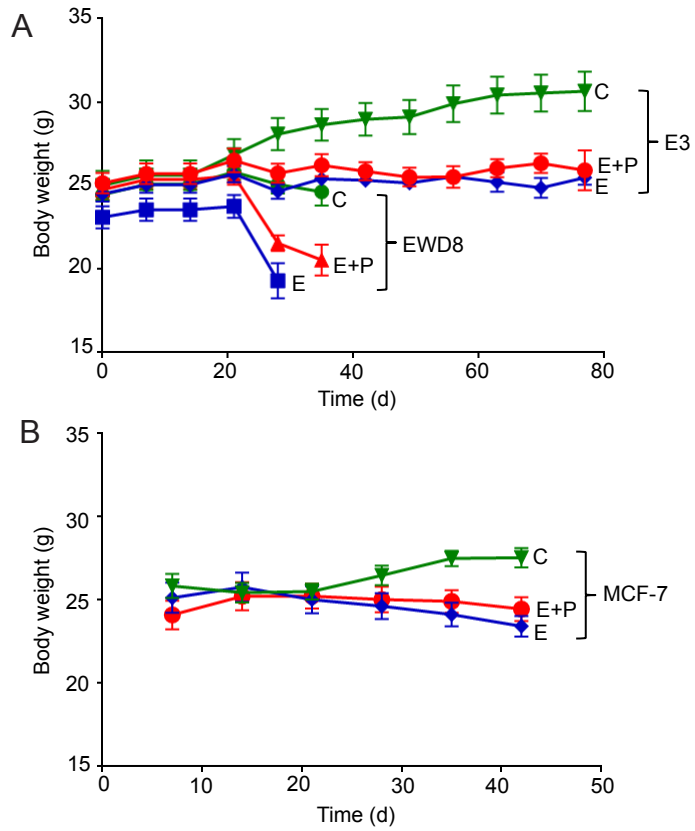

Supplement: Supplementary file 3 — Additional file 3: Body weights of mice during observation for development of metastases (A). Total body weights were measured weekly for ovariectomized (ovx’d) mice intracardiac (IC)-injected with estrogen withdrawn-line 8 (EWD8) or E3 cells treated with control (C), estrogen (E), or estrogen + progestin (E+P) for 80 days. Data are presented as mean ± standard error of the mean (SEM) (n = 20 per group). (B) Total body weights were measured weekly for ovx’d mice IC-injected with MCF-7 cells treated with C, E, or E+P for about 40 days. Data are presented as mean ± SEM (n = 20 per group). (PDF 218 KB) [file 13058_2014_489_MOESM3_ESM.pdf]

# Additional File 4

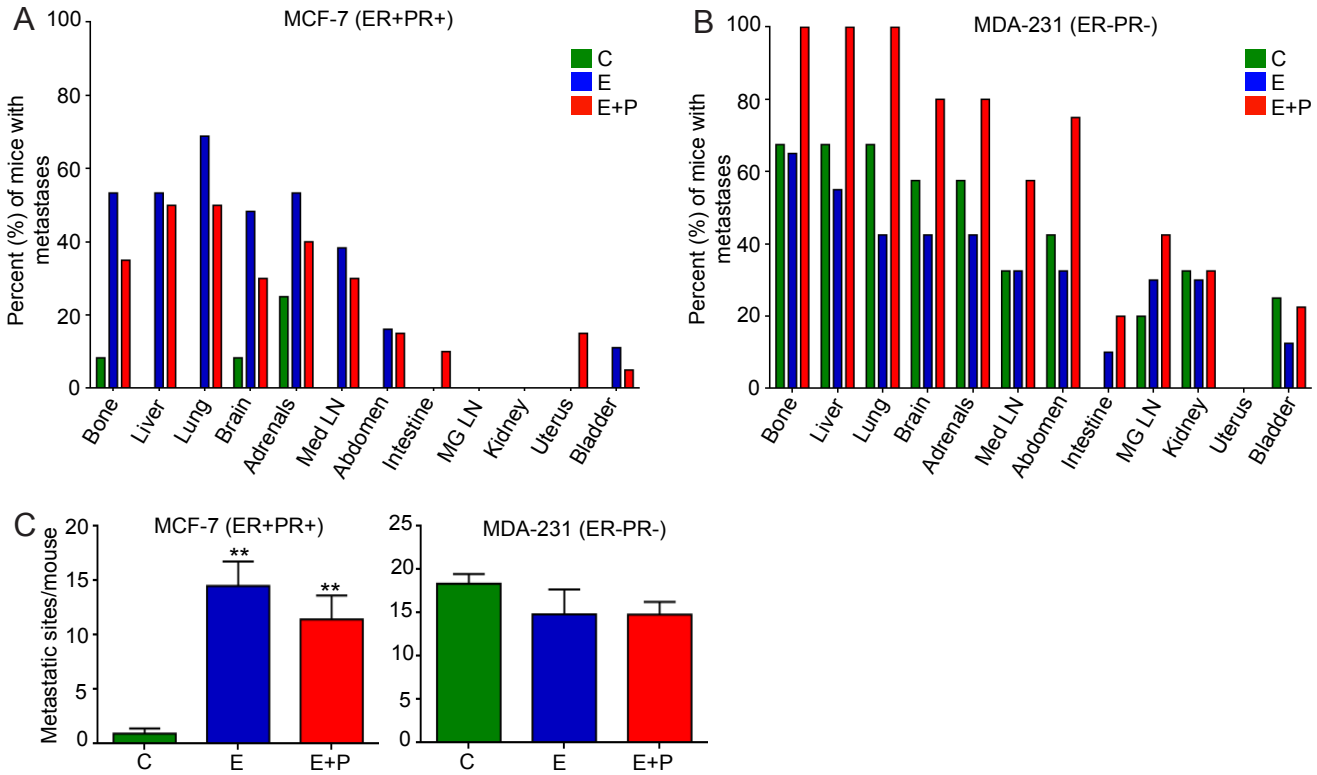

Supplement: Supplementary file 4 — Additional file 4: Hormonal regulation of MCF-7 and MDA-231 cell metastases. (A) Bar graph shows the percentage of mice in each treatment group—control (C) (green), estrogen (E) (blue), and estrogen + progestin (E+P) (red)—with MCF-7 metastases to distant organs. Data are presented as mean percentages per group (n = 20 per treatment group). (B) Bar graph shows the percentage of mice in each treatment group—C (green), E (blue), and E+P (red)—with MDA-231 metastases to distant organs. Data are presented as mean percentages per group (n = 10 per treatment group). (C) Number of ZsGreen-positive (ZsG+) metastatic sites per mouse intracardiac (IC)-injected with MCF-7 or MDA-MB-231 cells in C, E, or E+P states. Data are presented as mean ± standard error of the mean (SEM) (n = 20 per group for MCF-7; n = 10 per group for MDA-231). **P <0.005, Student’s t test. (PDF 239 KB) [file 13058_2014_489_MOESM4_ESM.pdf]

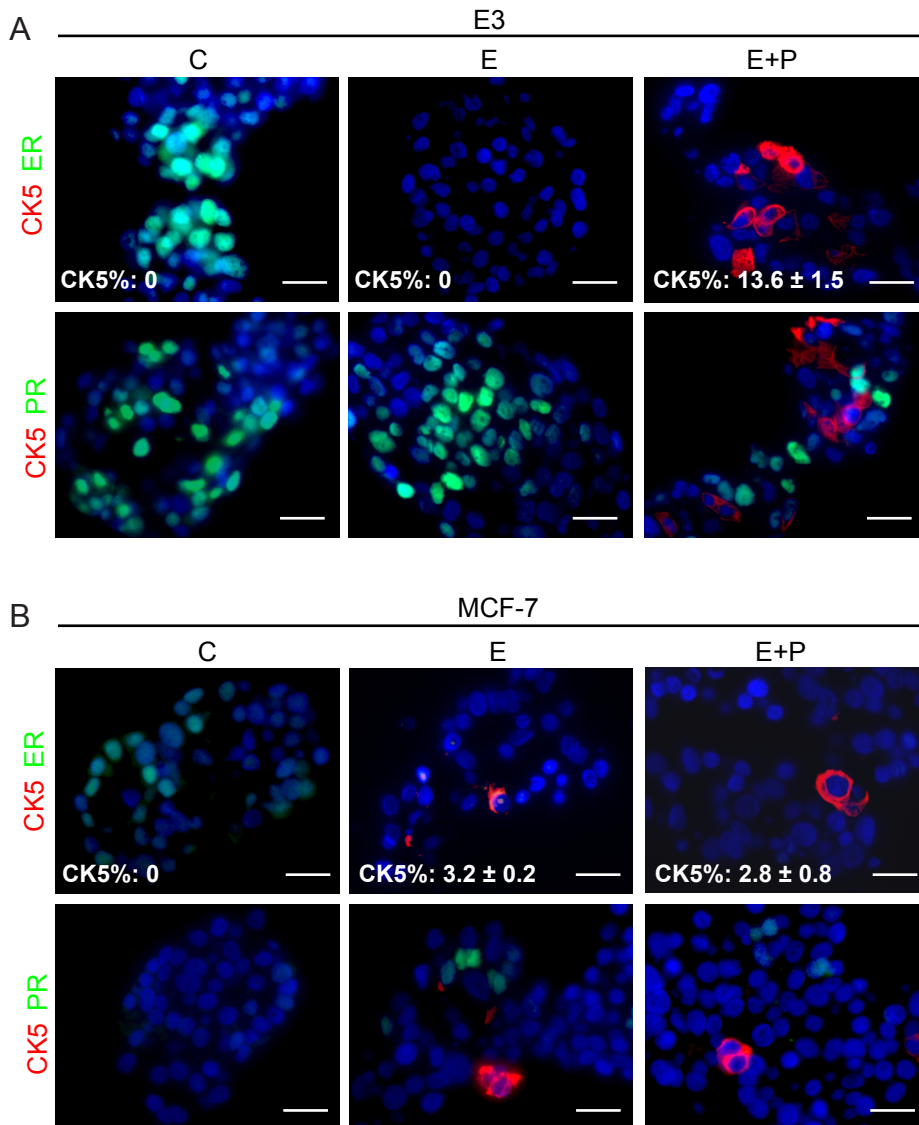

Supplement: Supplementary file 5 — Additional file 5: Hormones increase the number of cytokeratin 5-positive (CK5 +) cells in three-dimensional (3D) luminal colonies. (A) E3 cells were grown as 3D colonies in phenol red-free growth factor-reduced Matrigel and treated with control (ethanol, 1:1,000 vol/vol), 10 nM estrogen (E), or 100 nM progestin (P) for 1 week. Percentages of CK5+ cells are presented as mean ± standard error of the mean (SEM) values. Scale bars: 50 μm. (B) MCF-7 cells were grown as 3D colonies in phenol red-free growth factor-reduced Matrigel and treated with control, 10 nM E, and 100 nM P for 1 week. Percentages of CK5+ cells are presented as mean ± SEM values. Scale bars: 50 μm. Both images are representative of three independent experiments. (PDF 3 MB) [file 13058_2014_489_MOESM5_ESM.pdf]

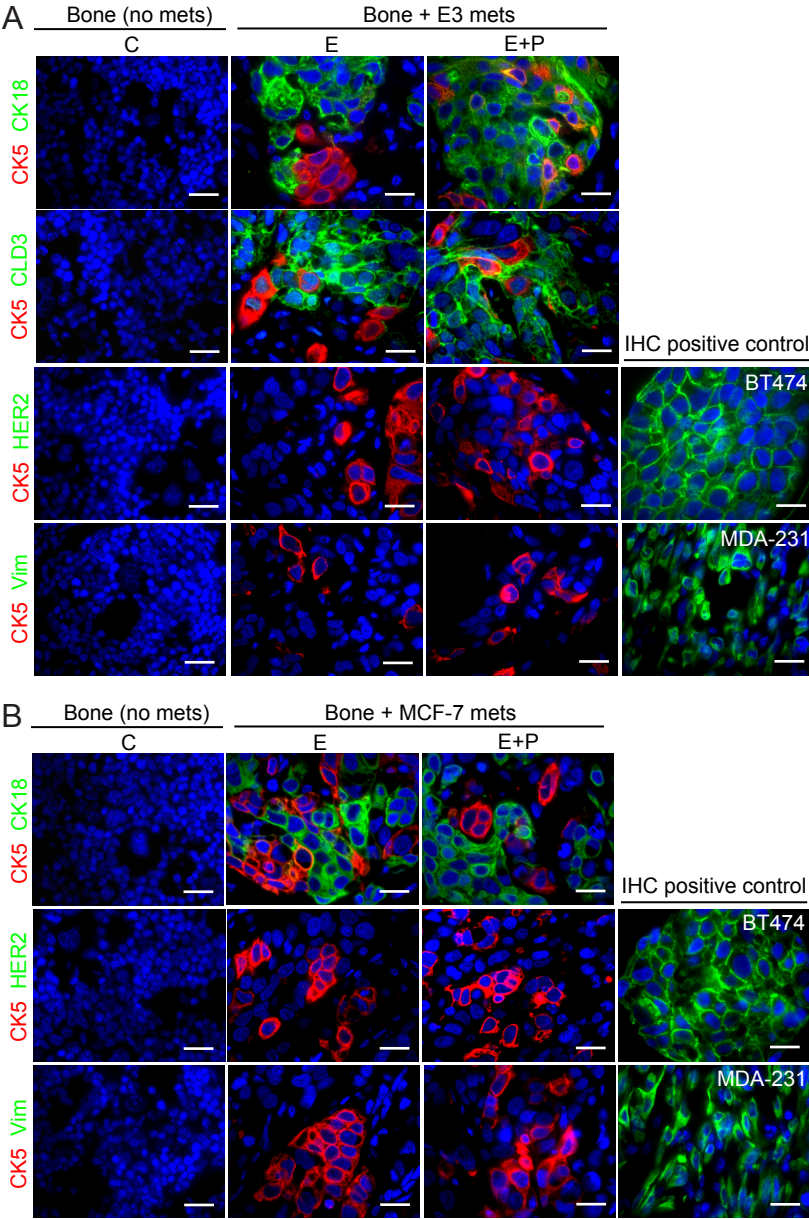

Supplement: Supplementary file 6 — Additional file 6: Immunohistochemistry (IHC) of luminal E3 or MCF-7 bone metastases showing heterogeneity for receptors and cytokeratin 5 (CK5), plus other markers. (A) Ovariectomized (Ovx’d) mice were intracardiac (IC)-injected with E3 cells and treated with control (C), estrogen (E), or estrogen + progestin (E+P). IHC: Bone sections were stained for CK8/18, CLD3, or HER2 (green); CK5 (red); and 4′,6-diamino-2-phenylindole (DAPI) counterstain (blue). Representative images are shown (n = 4 per group). BT474 and MDA-MB-231 cells were used as positive controls for HER2 or vimentin. Scale bars: 20 μm. (B) Ovx’d mice were IC-injected with MCF-7 cells and treated with C, E, or E+P. IHC: Bone sections were stained for CK8/18 or HER2 (green), CK5 (red), and DAPI (blue). Representative images are shown (n = 3 per group). BT474 and MDA-MB-231 cells were used as positive controls. Scale bars: 20 μm. (PDF 9 MB) [file 13058_2014_489_MOESM6_ESM.pdf]

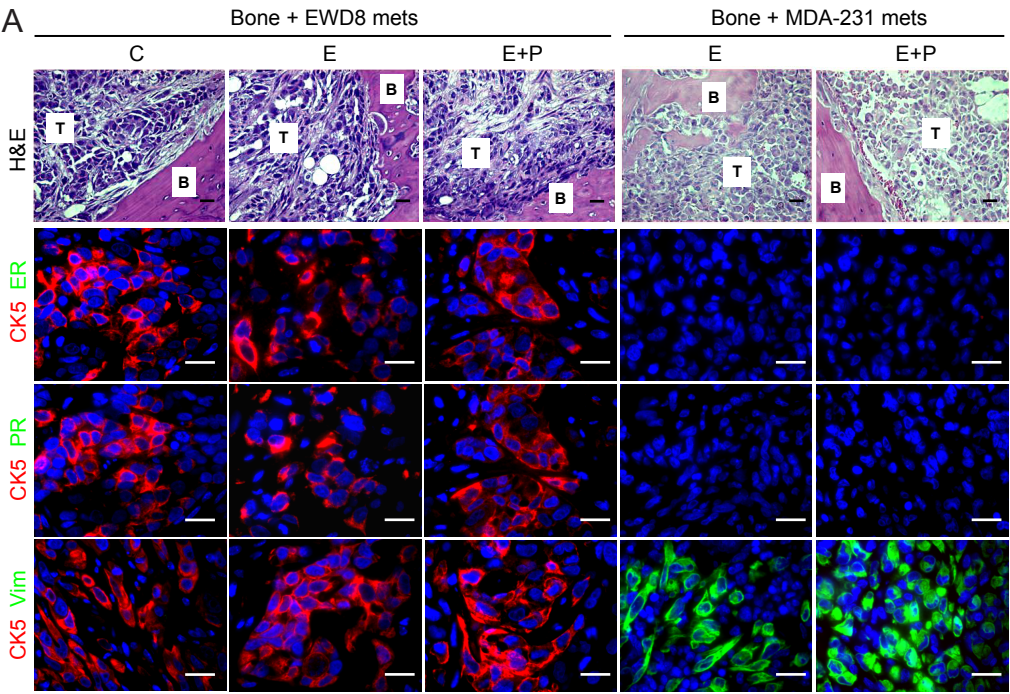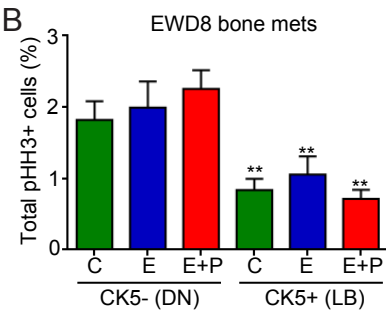

Supplement: Supplementary file 7 — Additional file 7: Heterogeneity of marker expression in basal-like estrogen withdrawn-line 8 (EWD8) or MDA-231 bone metastases. (A) Representative hematoxylin and eosin (H&E) and immunohistochemistry (IHC) of EWD8 or MDA-MB-231 bone metastases in mice treated with control (C), estrogen (E), or estrogen + progestin (E+P). H&E: bone (B), tumor (T). Scale bars: 50 μm. IHC: Dual staining for ER, PR, or vimentin (green), CK5 (red), and 4′,6-diamino-2-phenylindole (DAPI) (blue) is indicated (n = 5 per group). Scale bars: 20 μm. (B) Proliferation rate of luminobasal (LB) ER+PR+CK5+ and double-negative (DN) ER−PR−CK5− subpopulations measured with phosphor-histone H3 (pHH3) in C, E, and E+P-treated EWD8 bone metastases. Mean ± standard error of the mean (SEM) values are shown (n = 4 per group). **P <0.005, Student’s t test. (PDF 7 MB) [file 13058_2014_489_MOESM7_ESM.pdf]

Additional File 8

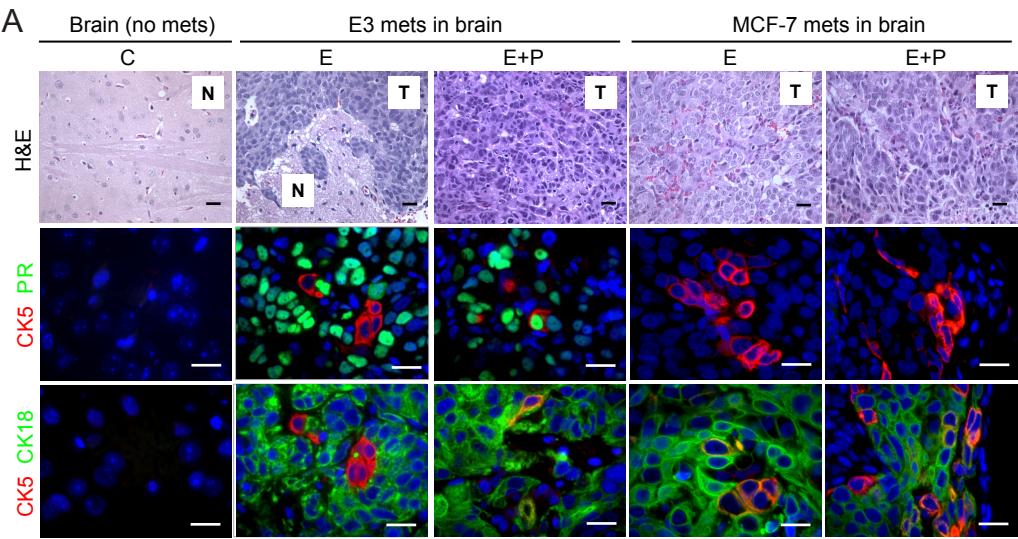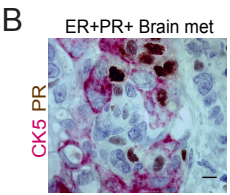

Supplement: Supplementary file 8 — Additional file 8: Heterogeneity of luminal brain metastases. (A) Hematoxylin and eosin (H&E) and immunohistochemistry (IHC) of E3 and MCF-7 brain metastases in mice treated with control (C), estrogen (E), or estrogen + progestin (E+P). H&E: Tumor-free or normal brain (N), tumor cells (T). Scale bars: 50 μm. IHC: Dual staining for progesterone receptor (PR) or cytokeratin 8/18 (CK8/18) (green), CK5 (red), and 4′,6-diamino-2-phenylindole (DAPI) (blue) (n = 3 per group). Scale bars: 20 μm. (B) Paraffin sections of brain metastases from a patient with luminal breast cancer; dual colorimetric staining for CK5 (pink) and PR (brown). Scale bars: 20 μm. (PDF 8 MB) [file 13058_2014_489_MOESM8_ESM.pdf]

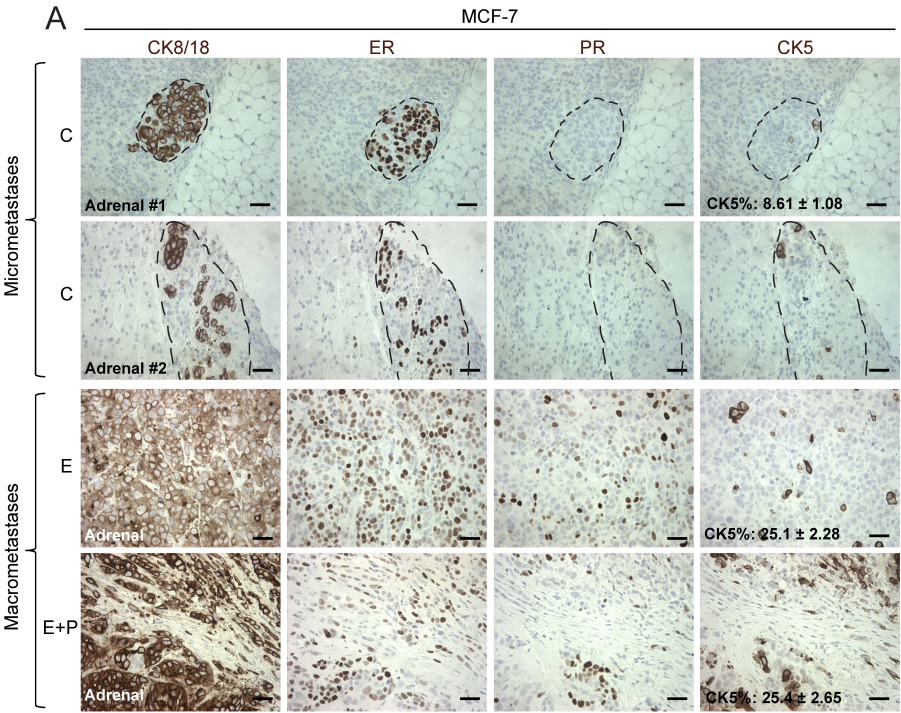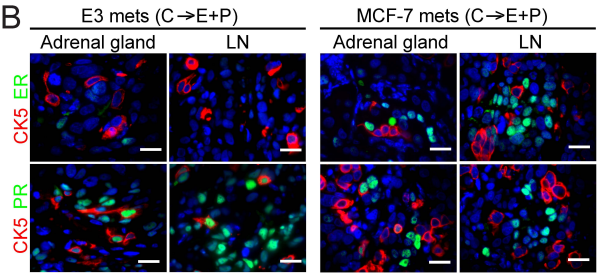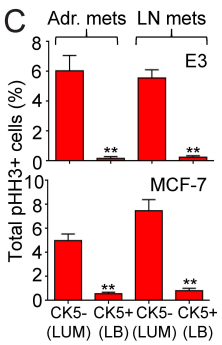

Supplement: Supplementary file 9 — Additional file 9: Hormones increase progesterone receptor (PR) and cytokeratin 5 (CK5) expression in MCF-7 macrometastases, but CK5 +cells are relatively quiescent. (A) Immunohistochemistry (IHC) for CK8/18, estrogen receptor (ER), PR, and CK5 in MCF-7 adrenal gland micrometastases formed in the absence of hormones (C), versus macrometastases formed with estrogen (E) and estrogen + progestin (E+P). Percentages of CK5+ cells are presented as mean ± standard error of the mean (SEM) values (n = 3 per group). Scale bars: 50 μm. (B) IHC for ER or PR (green), CK5 (red), and 4′,6-diamino-2-phenylindole (DAPI) (blue) of E3 and MCF-7 macrometastases in adrenals or LNs (n = 5 per group). Scale bars: 20 μm. (C) Proliferation rates of CK5+ and CK5− cells measured by phosphor-histone H3-positive (pHH3+) in E+P-treated E3 or MCF-7 adrenal (adr.) gland and LN metastases aroused from dormancy. Mean ± SEM values are presented (n = 3 per group). **P <0.005, Student’s t test. (PDF 3 MB) [file 13058_2014_489_MOESM9_ESM.pdf]

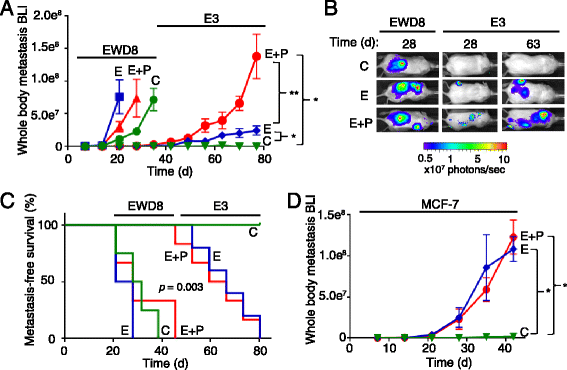

Supplement: Supplementary file 10 — Authors’ original file for figure 1 [file 13058_2014_489_MOESM10_ESM.gif]

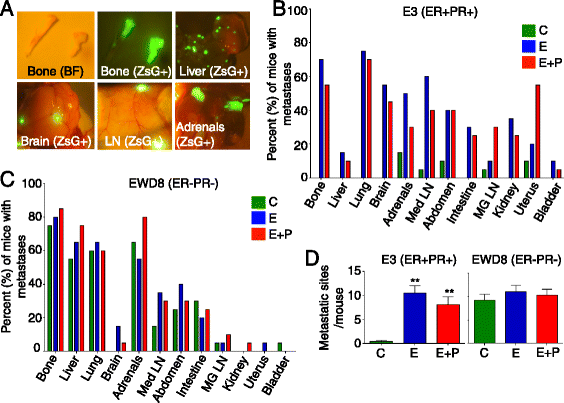

Supplement: Supplementary file 11 — Authors’ original file for figure 2 [file 13058_2014_489_MOESM11_ESM.gif]

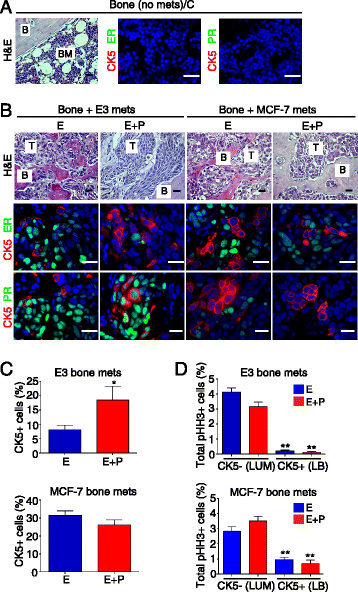

Supplement: Supplementary file 12 — Authors’ original file for figure 3 [file 13058_2014_489_MOESM12_ESM.gif]

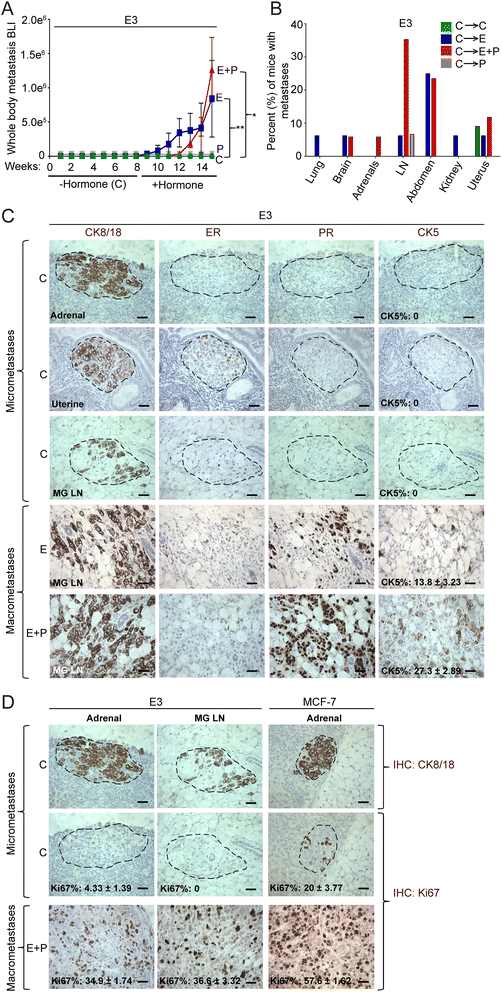

Supplement: Supplementary file 13 — Authors’ original file for figure 4 [file 13058_2014_489_MOESM13_ESM.gif]
